# Increased glycated albumin and decreased esRAGE levels in serum are related to negative coronary artery remodeling in patients with type 2 diabetes: an Intravascular ultrasound study

**DOI:** 10.1186/s12933-018-0792-y

**Published:** 2018-11-27

**Authors:** Run Du, Rui Yan Zhang, Lin Lu, Ying Shen, Li Jin Pu, Zheng Bin Zhu, Qi Zhang, Jian Hu, Zhen Kun Yang, Feng Hua Ding, Jian Sheng Zhang, Wei Feng Shen

**Affiliations:** Department of Cardiology, Rui Jin Hospital, Jiao Tong University School of Medicine, Shanghai, 200025 People’s Republic of China

**Keywords:** Coronary remodeling, Intravascular ultrasound, esRAGE, Glycated albumin, Diabetes mellitus

## Abstract

**Background:**

Negative coronary artery remodeling is frequent in patients with diabetes, but its mechanism remains unclear. We here evaluated the association of serum levels of glycated albumin (GA) and endogenous secretory receptor for advanced glycation end products (esRAGE) with coronary artery remodeling in type 2 diabetic patients.

**Methods:**

Serum levels of GA and esRAGE were measured and intravascular ultrasound was performed in 136 consecutive diabetic patients with 143 coronary intermediate lesions. The remodeling index (RI) was calculated as the ratio between external elastic membrane (EEM) area at the lesion site and EEM area at the reference segment. Negative remodeling (NR) was defined as an RI < 0.95 and intermediate or positive remodeling as an RI ≥ 0.95.

**Results:**

Mean plaque burden at the lesion site was 70.96 ± 9.98%, and RI was 0.96 ± 0.18. Negative coronary arterial remodeling existed in 81 (56.6%) lesions. RI correlated closely with serum esRAGE level (r = 0.236, P = 0.005) and was inversely related to serum GA level (r = − 0.240, P = 0.004) and plasma low-density lipoprotein cholesterol (LDL-C) (r = − 0.206, P = 0.014) and total cholesterol levels (r = − 0.183, P = 0.028). Generalized estimating equations logistic regression analysis identified esRAGE (OR 0.037; 95% CI 0.012–0.564, P = 0.021), GA (OR 1.093; 95% CI 1.013–1.179, P = 0.018) and LDL-C (OR 1.479; 95% CI 1.072–2.835, P = 0.023) as independent predictors for negative remodeling.

**Conclusions:**

In diabetic patients, negative coronary artery remodeling is associated with increased GA and decreased esRAGE levels in serum.

## Introduction

Coronary arterial remodeling (changes in vascular dimensions) occurs frequently during the development and progression of atherosclerosis, and is associated with clinical presentations in patients with coronary artery disease [1]. Multiple underlying pathophysiological determinants for the patterns and degree of coronary arterial remodeling have been recognized [2–4]. Patients with diabetes are prone to have an early and accelerated course of coronary atherosclerosis [5] and exhibit a markedly increased incidence of adverse cardiovascular events and less favorable outcomes after myocardial infarction or percutaneous coronary intervention (PCI) [6–9]. Intravascular ultrasound (IVUS) studies have shown that apart from lesion progression, coronary arteries in diabetics, compared with those in non-diabetics, are typically described as smaller and more likely to be diffuse and undergo negative remodeling (when the vascular area decreased as plaque develops) [10]. However, the exact mechanisms of negative coronary artery remodeling in diabetic patients remain not fully elucidated.

Interaction between advanced glycation end products (AGEs) produced in hyperglycemic milieu and their receptor (the receptor for AGEs, RAGE) plays an important role in the development of diabetic vascular complications [11–15]. Activation of RAGE-mediated pathways could lead to a series of adverse effects including impaired vascular homeostasis, enhanced inflammatory signaling and increased cellular oxidative stress. Glycated albumin (GA), an Amadori-modified early glycation product, intensifies inflammatory reaction and is associated with coronary artery disease in patients with diabetes [16–18]. In contrast, endogenous secretory RAGE (esRAGE), a novel splice variant carrying extracellular domains of RAGE but lacking transmembrane and cytoplasmic domains, acts as a protective factor for vascular function by antagonizing RAGE signaling via binding ligands including AGEs [19]. In this study, we sought to evaluate the association of increased GA and decreased esRAGE levels in serum with the incidence and degree of negative coronary artery remodeling in type 2 diabetic patients.

## Methods

### Study population

A total of 136 consecutive patients with type 2 diabetic mellitus who had at least one intermediate lesion (visually ascertained luminal diameter narrowing from 50 to 70% based on angiography) in a non-PCI major epicardial coronary artery from Shanghai Rui Jin Hospital PCI Outcome program between June 2009 and August 2010 were screened. Although the culprit lesions (diameter stenosis > 70%) absolutely represent disease status of patients, they had to be excluded from this study. The use of balloon to pre-dilate the culprit lesions might change the morphology of plaque and the pattern of lesion remodeling if IVUS catheter could not pass the lesions with severe stenosis.

The diagnosis of type 2 diabetes mellitus (T2DM) was made according to the criteria of American Diabetes Association (symptoms of diabetes with casual plasma glucose concentration ≥ 200 mg/dl [11.1 mmol/l] or fasting blood glucose (FBG) ≥ 126 mg/dl [7.0 mmol/l], 2 h postprandial glucose (2 h PG) ≥ 200 mg/dl [11.1 mmol/l] during an oral glucose tolerance test, and currently or previously treated with insulin and/or oral hypoglycemic agents) [20]. Hyperlipidemia was diagnosed in patients with total cholesterol levels of 200 mg/dl or higher and/or low-density lipoprotein (LDL) cholesterol levels of 130 mg/dl or higher and/or triglyceride values of 150 mg/dl or higher and/or high-density lipoprotein (HDL) cholesterol levels below 40 mg/dl or in patients with a present history of anti-hyperlipidemia drug use [21]. For the purpose of the study, we excluded these lesions with non-uniform rotational distortion which may cause IVUS artifacts, severely calcified lesions (arc of calcium > 90°) and ostial lesions. The study protocol was approved by hospital Ethics Committee and all patients provided written informed consent.

### IVUS imaging

IVUS imaging was performed after intracoronary administration of nitroglycerin (200 µg) with a motorized transducer pullback system and a commercial scanner (Galaxy or iLab; Boston Scientific, Natick, Massachusetts, USA) consisting of a rotating 30 or 40-MHz transducer. The ultrasound catheter was advanced 10 mm distal to the lesion, and transducer was withdrawn at 0.5 mm/s back at least 10 mm beyond the lesion or to the aorto-ostial junction. All real-time images were recorded on 0.5-inch discs for subsequent analysis.

IVUS imaging analysis was made by a single cardiologist (R.D.); the adequacy of the images was determined before unblinding the data for analysis. The target lesion and both proximal and distal reference segments were chosen for quantitative assessment. The lesion site was the image slice with smallest lumen cross-sectional area (CSA). The proximal and distal reference segments were most normal-looking segments with largest lumen and smallest plaque burden within 10 mm proximal and distal to the lesion with no major intervening branches. For each image slice, external elastic membrane (EEM) and lumen CSA were determined with planimetry software (QIvus, Medis, Netherlands). Plaque and media (P&M) CSA was calculated as EEM CSA minus lumen CSA. Plaque burden was calculated as EEM CSA divided by P&M CSA [22, 23].

**Fig. 1:**
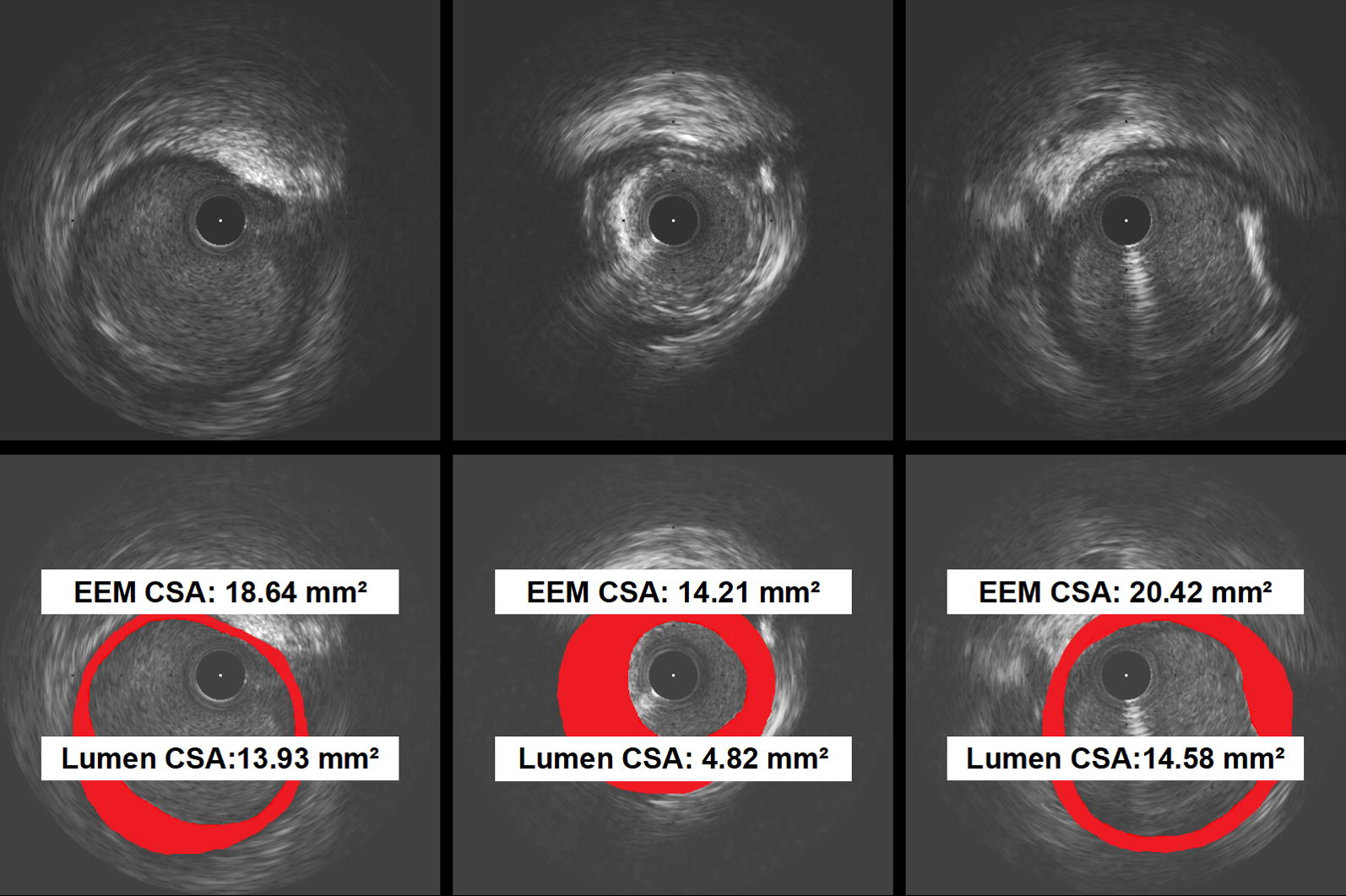
Measurements of coronary artery remodeling

Remodeling index (RI) was defined as lesion EEM CSA divided by mean reference EEM CSA (the average of the proximal and distal reference segments). Positive remodeling was defined as an RI > 1.05; intermediate remodeling was defined as an RI between 0.95 and 1.05; and negative remodeling as an RI < 0.95 [24] (Fig. 1). The lesions were classified into two groups according to the RI: negative remodeling (NR) group with RI < 0.95 [25]; and intermediate or positive remodeling (IR/PR) group with RI ≥ 0.95. Plaque composition was defined as soft, hard, or calcified according to the acoustic signal that arises from plaque in IVUS, following previously described guidelines [26]. At the lesion site, maximal and minimal thickness of the plaque was measured, and atheroma eccentricity index (EI) was calculated as maximum minus minimum plaque thickness divided by maximum plaque thickness. Concentric plaque was defined as EI < 0.5, and eccentric plaque as EI ≥ 0.5 [27].

### Biochemical investigation

Blood samples were collected after overnight fasting and stored at − 80°. Plasma levels of glucose and lipid profiles including total cholesterol (TC), low-density lipoprotein cholesterol (LDL-C), high-density lipoprotein cholesterol (HDL-C), lipoprotein (a) (Lp (a)), apoprotein-A and -B (Apo-A and -B), and triglycerides (TG) were measured (HITACHI 912 Analyser, Roche Diagnostics, Germany). Serum GA levels were determined with an improved bromocresolpurple method using the lucica™ glycated albumin-L assay kit (Asahi Kasei Pharma, Japan). The linear range for this assay was 3.2–68.1% and the maximum inter-assay coefficient of variation was < 3.0%. Serum levels of esRAGE were determined by an ELISA kit (Phoenix Biotech Co, LTD, USA), which specifically measures esRAGE but not c-RAGE as detailed in protocol provided by the manufacturer. The linear range of the measurement and interassay coefficient of variation for esRAGE were 0.05–3.2 ng/mL and < 10%, respectively.

### Statistical analysis

Statistical analysis was performed using the SPSS statistical package, version 13.0 (SPSS Inc, USA). Continuous variables were presented as mean ± SD and compared by one-way ANOVA with post hoc analysis in two-group comparisons using Fisher PLSD or Dunnett’s T3 tests. Categorical variables were presented as frequencies and compared with Chi square test or Fisher’s exact test. The strength of association between two continuous variables was evaluated with Pearson correlation analysis or Spearman rank order correlation test, as appropriate. Using a 2-sided 5% significance level, we estimated that total 113 patients were needed with correlation coefficient of 0.3 and a statistical power of 90%. To identify independent predictors of NR, logistic regression with generalized estimating equations (GEE) method was used accounting for the within-subject correlation due to multiple lesions analyzed within the same patient. Those variables associated with RI with a P-value less than 0.05 in the univariate analysis or which were clinically relevant were entered into the multivariate model. All tests of significance were two-tailed and a P < 0.05 was considered significant.

## Results

### Baseline characteristics

A total of 136 diabetic patients with 143 lesions were enrolled in this study. Baseline characteristics are presented in Table 1. Mean plaque burden at the lesion site was 70.96 ± 9.98%, and overall, RI was 0.96 ± 0.18. 81 lesions (56.6%) had negative remodeling (Table 2).

**Table 1:** Baseline characteristics

Characteristics (N = 136 DM patients)	
Age (years)	65.3 ± 9.28
Women n, (%)	53 (39.0)
Body mass index (kg/m^2^)	26.94 ± 3.01
Blood pressure (mmHg)	
Systolic blood pressure	133.34 ± 15.66
Diastolic blood pressure	78.98 ± 8.82
Risk factors	
Hypertention n, (%)	102 (75.0)
Hyperlipidemia n, (%)	78 (57.4)
Smoking n, (%)	54 (39.7)
LVEF (%)	63.79 ± 6.28
Clinical presentation	
Stable angina n, (%)	45 (33.1)
Unstable angina n, (%)	63 (46.3)
Acute myocardial infarction n, (%)	24 (17.7)
Old myocardial infarction n, (%)	4 (2.9)
Target vessels n (%)	
LAD	77 (53.8)
LCX	20 (14.0)
RCA	46 (32.2)
Laboratory study	
Total cholesterol (mmol/L)	4.27 ± 1.00
Triglycerides (mmol/L)	2.18 ± 1.27
HDL-C (mmol/L)	1.08 ± 0.25
LDL-C (mmol/L)	2.42 ± 0.85
Apo-A (mmol/L)	1.13 ± 0.18
Apo-B (mmol/L)	0.90 ± 0.22
Lp (a) (g/L)	0.19 ± 0.15
Fast glucose (mmol/L)	7.55 ± 2.50
BUN (mmol/L)	5.98 ± 1.65
Creatinine (mg/L)	86.19 ± 24.58
Glycated haemoglobin (%)	7.84 ± 1.51
hsCRP (mg/L)	5.47 ± 6.11
esRAGE (ng/ml)	0.20 ± 0.15
GA (%)	21.63 ± 5.54
Oral hypoglycemic agents n, (%)	112 (82.4)
Insulin n, (%)	31 (22.8)
Duration of DM treatment (years)	11.2 ± 8.5
Antiplatalet n, (%)	136 (100)
Statins n, (%)	132 (97.1)

DM, diabetes mellitus; LVEF, left ventricle ejection fraction; LAD, left anterior descending; LCX, left circumsflex; RCA, right coronary artery; HDL-C, high density lipoprotein-cholesterol; LDL-C, low density lipoprotein-cholesterol; Apo-A, apolipoprotein-A; Apo-B, apolipoprotein-B; Lp (a), lipoprotein (a); BUN, blood urea nitrogen; hsCRP, high sensitive C-reactive protein; esRAGE, endogenous secretory receptors for advanced glycation endproducts; GA, glycated albumin

**Table 2:** IVUS measurements

	NR group (N = 81)	IR/PR group (N = 62)	P value
Lesion site			
EEM CSA (mm^2^)	12.54 ± 4.39	16.50 ± 4.51	< 0.001
Lumen CSA (mm^2^)	3.91 ± 1.73	4.08 ± 1.76	0.564
P&M CSA (mm^2^)	8.63 ± 3.50	12.42 ± 4.04	< 0.001
Plaque burden (%)	68.08 ± 10.04	74.71 ± 8.62	< 0.001
Eccentricity index	0.77 ± 0.20	0.74 ± 0.20	0.423
Eccentric lesion, n (%)	70 (86.4)	54 (87.1)	0.906
Proximal reference			
EEM CSA (mm^2^)	15.67 ± 4.67	15.63 ± 3.76	0.949
Lumen CSA (mm^2^)	10.44 ± 3.62	9.75 ± 3.19	0.241
P&M CSA (mm^2^)	5.31 ± 2.97	5.87 ± 2.67	0.245
Plaque burden (%)	31.80 ± 16.39	35.88 ± 17.03	0.150
Distal reference			
EEM CSA (mm^2^)	14.32 ± 6.70	14.34 ± 4.76	0.983
Lumen CSA (mm^2^)	9.07 ± 3.28	9.05 ± 4.06	0.983
P&M CSA (mm^2^)	4.79 ± 3.65	5.29 ± 3.42	0.404
Plaque burden (%)	31.81 ± 15.68	35.81 ± 19.52	0.176
Plaque composition			
Soft, n (%)	18 (22.2)	19 (32.3)	0.178
Fibrous, n (%)	45 (55.6)	33 (53.2)	0.782
Calcific (< 90°), n (%)	13 (16.0)	7 (11.3)	0.416
Mixed, n (%)	5 (6.3)	3 (4.8)	0.718

NR, negative remodeling; IR/PR, intermediate or positive remodeling; CSA, cross sectional area; P&M, Plaque and media; EEM, external elastic membrane

### IVUS findings

There were no significant differences between NR group and IR/PR group with regard to parameters of proximal and distal reference vessels. The Eccentricity index was also similar in the two groups. At the culprit lesion site, NR group had smaller EEM CSA, P&M CSA and Plaque burden. No significant differences were observed in the plaque composition between the two groups (Table 2).

### Biochemical measurements

**Fig. 2:**
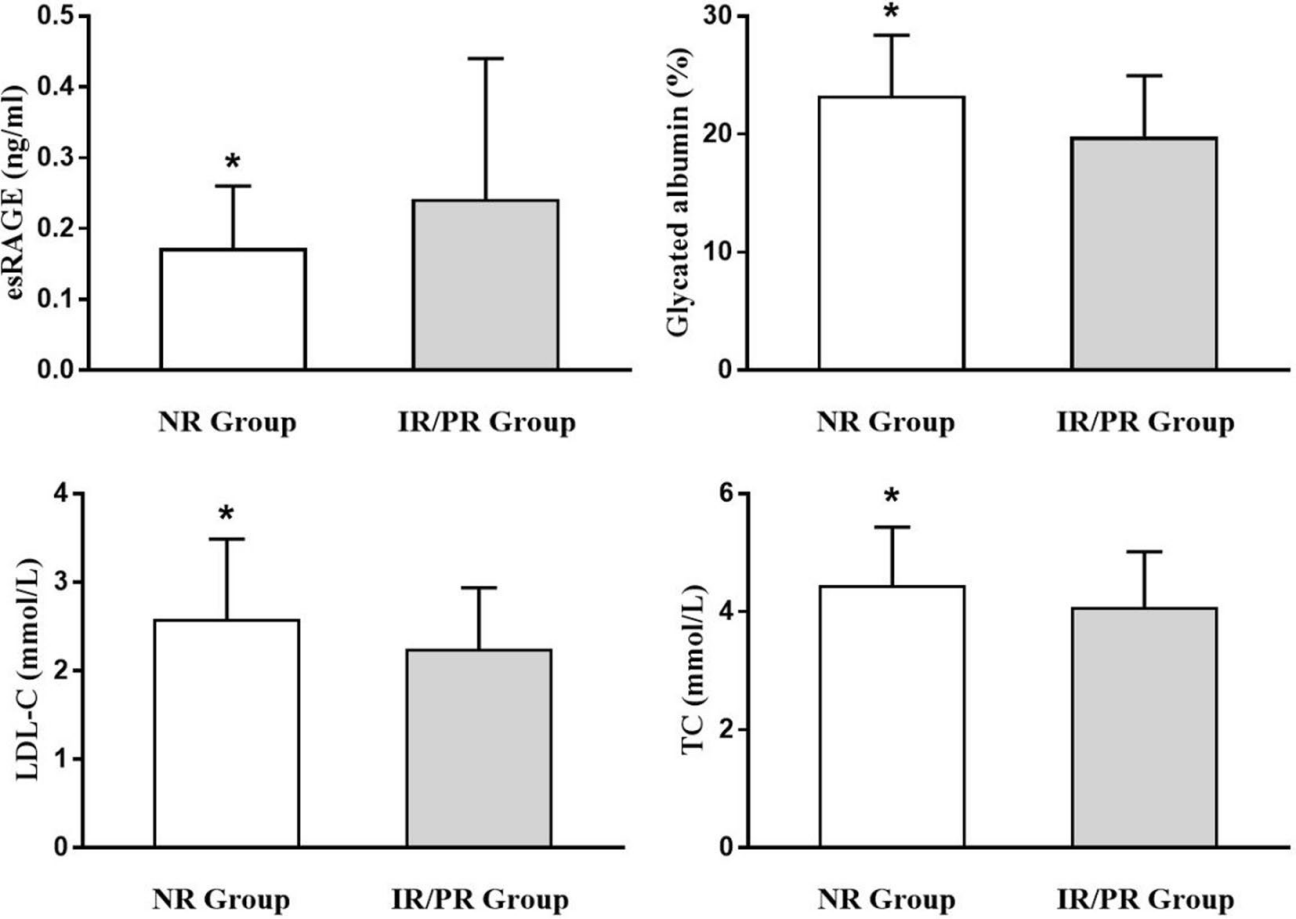
Comparison of serum levels of esRAGE, glycated albumin, LDL-C and total cholesterol (TC) between negative remodeling (NR) and intermediate or positive remodeling (IR/PR) groups. *P < 0.05 vs. IR/PR group

Metabolic profiles were impaired in lesions with negative remodeling with lower levels of esRAGE and higher levels of GA, LDL-C and total cholesterol when compared with lesions with RI ≥ 0.95 (Table 3) (Fig. 2).

**Table 3:** Biochemical assessments

	NR group (N = 81)	IR/PR group (N = 62)	P value
Total cholesterol (mmol/L)	4.43 ± 1.00	4.06 ± 0.96	0.029
Triglycerides (mmol/L)	2.11 ± 1.06	2.26 ± 1.51	0.498
HDL-C (mmol/L)	1.10 ± 0.26	1.06 ± 0.23	0.331
LDL-C (mmol/L)	2.57 ± 0.92	2.23 ± 0.71	0.017
Apo-A (mmol/L)	1.14 ± 0.18	1.11 ± 0.18	0.330
Apo-B (mmol/L)	0.90 ± 0.22	0.90 ± 0.22	0.902
Lp (a)	0.19 ± 0.11	0.19 ± 0.20	0.873
Fast glucose (mmol/L)	7.67 ± 2.82	7.38 ± 2.02	0.489
BUN (mmol/L)	5.94 ± 1.52	6.04 ± 1.82	0.718
Creatinine (mg/L)	84.84 ± 18.74	87.96 ± 30.66	0.454
Glycated haemoglobin (%)	7.92 ± 1.61	7.73 ± 1.39	0.451
hsCRP (mg/L)	5.33 ± 5.35	5.67 ± 7.02	0.745
esRAGE (ng/mL)	0.17 ± 0.09	0.24 ± 0.20	0.003
GA (%)	23.15 ± 5.24	19.64 ± 5.33	< 0.001

NR, negative remodeling; IR/PR, intermediate or positive remodeling; HDL-C, high density lipoprotein-cholesterol; LDL-C, low density lipoprotein-cholesterol; Apo-A, apolipoprotein-A; Apo-B, apolipoprotein-B; Lp (a), lipoprotein (a); BUN, blood urea nitrogen; hsCRP, high sensitive C-reactive protein; esRAGE, endogenous secretory receptors for advanced glycation endproducts; GA, glycated albumin

### Predictors of remodeling

**Fig. 3:**
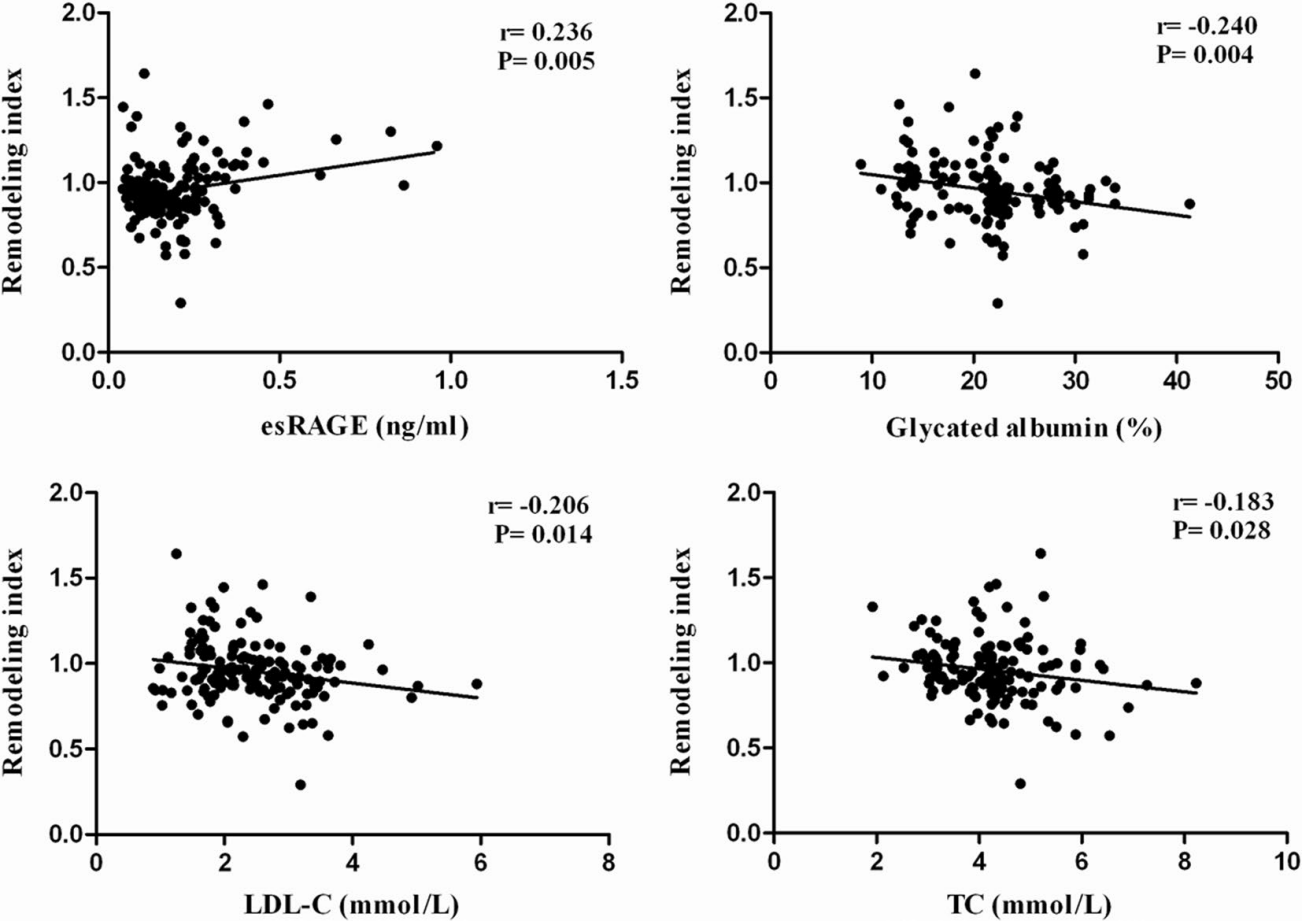
Correlation of remodeling index (RI) with serum levels of esRAGE, glycated albumin, LDL-C and total cholesterol (TC)

No significant correlation of RI with clinical findings and plaque composition were found in the two groups by univariate analysis. RI positively correlated with serum level of esRAGE (r = 0.236, P = 0.005) and was inversely related to GA (r = − 0.240, P = 0.004), LDL-C (r = − 0.206, P = 0.014) and total cholesterol (r = − 0.183, P = 0.028) (Fig. 3). GEE logistic regression analysis identified esRAGE (OR 0.037; 95% CI 0.012–0.564, P = 0.021), GA (OR 1.093; 95% CI 1.013–1.179, P = 0.018) and LDL-C (OR 1.479; 95% CI 1.072–2.835, P = 0.023) as independent predictors for negative remodeling.

## Discussion

The current study is the first to show increased GA and decreased esRAGE levels in serum aggravate RAGE- and, possibly, non-RAGE-mediated vascular damage, resulting in coronary artery negative remodeling in type 2 diabetic patients.

### Coronary artery remodeling and incidence in diabetes

Coronary artery remodeling, initially described by Glagov et al. [28], is a common adaptive vessel response during the process of atherosclerosis. The outer wall of the artery, encompassed by EEM, enlarges to accommodate the atherosclerotic plaque growth, thus the lumen size could be maintained. However, such a compensatory vascular enlargement (positive remodeling) does not usually occur, but conversely, coronary arterial shrinkage (negative remodeling) is prevalent in diabetes [29]. In a pooled analysis of five IVUS trials, diabetic patients demonstrated a greater atheroma volume and exhibited a smaller lumen with no differences in EEM compared with non-diabetic counterparts [30]. Likewise, negative coronary artery remodeling occurs in about 72% of lesions in the DIABETES trial [10]. In this study, we observed that almost two-thirds of patients with type 2 diabetes developed negative coronary artery remodeling, which substantiated the concept that compensatory positive remodeling was impaired in diabetic patients, and constrictive remodeling might itself be attributed to an inability for adaptive remodeling.

### The underlying determinants of coronary artery remodeling

The mechanism that induces arterial remodeling in diabetes is likely to be complex. Positive remodeling is commonly associated with vulnerable plaque characterized by greater plaque volume, necrotic core [31] and thinning change of the fibrous cap [32]. In diabetic patients, advanced plaque phenotype and further atherosclerosis progression could still be found despite lipid-lowering therapy [33]. Worse glucose control and higher blood glucose variability were documented to independently increase lipid and decrease fibrous contents with larger plaque burden and necrotic core volume through various mechanisms [34–39]. Renal function could also contribute the effects of blood glucose fluctuations and blood levels of inflammatory cytokine concentrations on the tissue characteristics of coronary plaques in diabetic patients [40]. In addition, increased insulin resistance (IR) was proved to be significantly associated with a higher remodeling index and positive coronary artery remodeling [41]. The underlying pathophysiological determinants which promote impaired arterial remodeling in diabetes remain unclear. It is possible that increasing deposition of fibrous and calcific tissue in the arterial wall, in addition to impaired endothelial-dependent relaxation [42, 43], may limit vessel wall expansion with plaque accumulation. Previous studies including ours showed that serum LDL level was independently related to negative coronary artery remodeling in diabetic patients [10]. In this study, we found that serum GA levels were elevated but serum esRAGE levels were decreased in diabetic patients with RI < 0.95. Multivariate analysis identified GA and esRAGE as independent predictors for negative remodeling.

### GA/esRAGE and negative remodeling

GA may reflect, to some extent, the severity of cross-link of collagen caused by AGEs in extracellular matrix. The exposure of GA of proximal tubular cells may also induce a decrease of metalloproteinase activities and an subsequent increase of secreted collagen (type I, III, and IV) [44, 45]. Non-enzymatic glycosylation of matrix proteins, especially collagen, induces protein cross-linking, which could lead to subsequent reduction in arterial elasticity [43]. AGEs may also inhibit synthesis and activity of matrix metalloproteinases, which are central to the definition of extracellular matrix composition, structure and remodeling, by accentuating production of growth factors and cytokines (such as transforming growth factor beta) in vascular smooth muscle cells (SMCs), thus reducing the degradation of collagen and hydrolyzation of elastin [46]. Clinical studies had documented that esRAGE and GA were significantly associated with arterial stiffness in type 2 diabetic patients [42] and in hemodialysis patients with type 2 diabetes [47]. The loss of arterial elasticity impairs the ability of artery wall to expand in response to accumulation of plaque, while further progression of protein cross-linking may cause vessel shrinkage. On the other hand, AGEs may impaire the endothelium-dependent vasorelaxation by significantly reducing endothelial nitric oxide synthase (eNOS) expression level and NOS activity as well as NO availability in coronary artery endothelial cells [48]. Furthermore, GA and decreased esRAGE independently contribute to vascular calcification [49, 50], which could reduce remodeling capacity, thus could predict subsequent decreased arterial remodeling [51]. Taken together, these results support an independent mechanism of elevated GA and decreased esRAGE in serum contributory to negative coronary artery remodeling in diabetic patients.

### Remodeling and clinical events

The relationship between negative remodeling and clinical events remain unclear. Vessel shrinkage in diabetes appeared as a late event in the development of atherosclerosis, thus appeared as the major determinant of lumen reduction during follow-up in nonsignificant stenotic plaques [10]. In patients with acute coronary syndrome (ACS), negative lesion site remodeling was documented to be associated with unanticipated nonculprit lesion major adverse cardiac events (MACE) [52]. However, compared with negative remodeling, culprit lesion positive remodeling in ACS was associated with a higher rate of adverse cardiac events. Acute myocardial infarction (AMI) patients with culprit lesion positive remodeling had more plaque vulnerability and higher frequency of plaque prolapse accompanied by post-procedural cardiac enzyme elevation than patients with negative remodeling [53]. During the long term follow-up, both target lesion revascularization (TLR) and non-TLR rates were significantly higher in ACS patients with positive remodeling than in those with negative remodeling [54]. Cardiac event-free survival was significantly lower in culprit lesion positive remodeling group than that in the IR/NR group. Culprit lesion PR, but not IR/NR, was associated with a poor long-term prognosis in patients with ACS [25].

### Potential clinical application

Circulating GA and esRAGE might be directly implicated as an independent mechanism, thus could serve as novel biomarkers in the development of coronary artery remodeling in diabetes. Previous animal studies showed that genetically engineered esRAGE and sRAGE could prevent the development of micro- [55] and macrovascular [56, 57] complications in diabetes. Currently available pharmacological agents were demonstrated to modulate the levels of esRAGE and sRAGE [58]. Given their role in inadequate arterial remodeling which could promote obstructive disease, the AGE-RAGE system could become potential target for therapeutic modification in diabetic patients with coronary artery disease.

## Limitations

Our study assessed coronary artery remodeling by comparing the lesion EEM to the reference segments at one time point, thus any conclusion about natural history of vascular remodeling of atherosclerotic plaques, during time in diabetic patients could not be drawn, and the relationship between negative remodeling and clinical events could also not be evaluated. The sample size was relatively small. We did not examine the coronary lesions by IVUS with angiographic diameter stenosis > 70% and < 50%, so the remodeling pattern and relationship of serum levels of GA and esRAGE with remodeling status in mild and severe stenotic lesions were not known. Serum GA and esRAGE levels may vary by race [59, 60]. Our findings may also not be applicable to overtly calcified lesions, as these lesions were excluded from this study considering the unreliable measurement of EEM area. While some inaccuracy of measurement of EEM area of plaques contacting deposits of calcium could not also be ruled out.

## Conclusions

This study demonstrates an association of increased GA levels and decreased esRAGE levels in serum with negative coronary artery remodeling in patients with type 2 diabetes mellitus.

## Abbreviations

AGEs: advanced glycation end products
AMI: acute myocardial infarction
CSA: cross-sectional area
CI: confidence interval
Apo-A and -B: apoprotein-A and -B
EEM: external elastic membrane
EI: eccentricity index
eNOS: endothelial nitric oxide synthase
esRAGE: endogenous secretory receptor for advanced glycation end products
FBG: fasting blood glucose
GA: glycated albumin
GEE: generalized estimating equations
HDL-C: high-density lipoprotein cholesterol
IR: insulin resistance
IR/PR: intermediate or positive remodeling
IVUS: intravascular ultrasound
LDL-C: low-density lipoprotein cholesterol
Lp (a): lipoprotein (a)
MACE: major adverse cardiac events
NR: negative remodeling
OR: odds ratios
PCI: percutaneous coronary intervention
P&M: plaque and media
2 hPG: 2h postprandial glucose
RAGE: the receptor for AGEs
RI: remodeling index
SMCs: smooth muscle cells
T2DM: type 2 diabetes mellitus
TC: total cholesterol
TG: triglycerides
TLR: target lesion revascularization

## References

[1] Nakamura M, Nishikawa H, Mukai S, Setsuda M, Nakajima K, Tamada H, Suzuki H, Ohnishi T, Kakuta Y, Nakano T, Yeung AC (2001). Impact of coronary artery remodeling on clinical presentation of coronary artery disease: an intravascular ultrasound study. J Am Coll Cardiol.

[2] Britten MB, Zeiher AM, Schächinger V (2003). Effects of cardiovascular risk factors on coronary artery remodeling in patients with mild atherosclerosis. Coron Artery Dis.

[3] Weissman NJ, Sheris SJ, Chari R, Mendelsohn FO, Anderson WD, Breall JA, Tanguay JF, Diver DJ (1999). Intravascular ultrasonic analysis of plaque characteristics associated with coronary artery remodeling. Am J Cardiol.

[4] Mintz GS, Kent KM, Pichard AD, Satler LF, Popma JJ, Leon MB (1997). Contribution of inadequate arterial remodeling to the development of focal coronary artery stenoses. An intravascular ultrasound study. Circulation..

[5] Kannel WB, McGee DL (1979). Diabetes and glucose tolerance as risk factors for cardiovascular disease: the Framingham study. Diabetes Care.

[6] Kip KE, Faxon DP, Detre KM, Yeh W, Kelsey SF, Currier JW (1996). Coronary angioplasty in diabetic patients. The national heart, lung, and blood institute percutaneous transluminal coronary angioplasty registry. Circulation.

[7] Huxley R, Barzi F, Woodward M (2006). Excess risk of fatal coronary heart disease associated with diabetes in men and women: meta-analysis of 37 prospective cohort studies. BMJ.

[8] Mehran R, Dangas GD, Kobayashi Y, Lansky AJ, Mintz GS, Aymong ED, Fahy M, Moses JW, Stone GW, Leon MB (2004). Short- and long-term results after multivessel stenting in diabetic patients. J Am Coll Cardiol.

[9] Kornowski R, Mintz GS, Kent KM, Pichard AD, Satler LF, Bucher TA, Hong MK, Popma JJ, Leon MB (1997). Increased restenosis in diabetes mellitus after coronary interventions is due to exaggerated intimal hyperplasia. A serial intravascular ultrasound study. Circulation..

[10] Jiménez-Quevedo P, Suzuki N, Corros C, Ferrer C, Angiolillo DJ, Alfonso F, Hernández-Antolín R, Bañuelos C, Escaned J, Fernández C, Costa M, Macaya C, Bass T, Sabaté M (2009). Vessel shrinkage as a sign of atherosclerosis progression in type 2 diabetes: a serial intravascular ultrasound analysis. Diabetes.

[11] Schmidt AM, Yan SD, Stern DM (1995). The dark side of glucose. Nat Med.

[12] Hofmann MA, Drury S, Fu C, Qu W, Taguchi A, Lu Y, Avila C, Kambham N, Bierhaus A, Nawroth P, Neurath MF, Slattery T, Beach D, McClary J, Nagashima M, Morser J, Stern D, Schmidt AM (1999). RAGE mediates a novel proinflammatory axis: a central cell surface receptor for S100/calgranulin polypeptides. Cell.

[13] McNair E, Qureshi M, Prasad K, Pearce C (2016). Atherosclerosis and the hypercholesterolemic AGE-RAGE axis. Int J Angiol.

[14] Yang ZK, Shen Y, Shen WF, Pu LJ, Meng H, Zhang RY, Zhang Q, Chen QJ, De Caterina R, Lu L (2015). Elevated glycated albumin and reduced endogenous secretory receptor for advanced glycation endproducts levels in serum predict major adverse cardio-cerebral events in patients with type 2 diabetes and stable coronary artery disease. Int J Cardiol.

[15] Saremi A, Howell S, Schwenke DC, Bahn G, Beisswenger PJ, Reaven PD (2017). VADT investigators. advanced glycation end products, oxidation products, and the extent of atherosclerosis during the va diabetes trial and follow-up study. Diabetes Care.

[16] Pu LJ, Lu L, Xu XW, Zhang RY, Zhang Q, Zhang JS, Hu J, Yang ZK, Ding FH, Chen QJ, Lou S, Shen J, Fang DH, Shen WF (2006). Value of serum glycated albumin and high-sensitivity C-reactive protein levels in the prediction of presence of coronary artery disease in patients with type 2 diabetes. Cardiovasc Diabetol..

[17] Ma X, Hu X, Zhou J, Hao Y, Luo Y, Lu Z, Bao Y, Jia W (2015). Glycated albumin is more closely correlated with coronary artery disease than 1,5-anhydroglucitol and glycated hemoglobin A1c. Cardiovasc Diabetol..

[18] Norimatsu K, Miura S, Suematsu Y, Shiga Y, Miyase Y, Nakamura A, Yamada M, Matsunaga A, Saku K (2015). Associations between glycated albumin or hemoglobin A1c and the presence of coronary artery disease. J Cardiol.

[19] Yonekura H, Yamamoto Y, Sakurai S, Petrova RG, Abedin MJ, Li H, Yasui K, Takeuchi M, Makita Z, Takasawa S, Okamoto H, Watanabe T, Yamamoto H (2003). Novel splice variants of the receptor for advanced glycation end-products expressed in human vascular endothelial cells and pericytes, and their putative roles in diabetes-induced vascular injury. Biochem J..

[20] Alberti KG, Definition Zimmet PZ (1998). diagnosis and classification of diabetes mellitus and its complications. Part 1: diagnosis and classification of diabetes mellitus provisional report of a WHO consultation. Diabet Med.

[21] Joint committee issued Chinese guideline for the management of dyslipidemia in adults (2016). Chinese guideline for the management of dyslipidemia in adults. Chin J Cardiol..

[22] Sabaté M, Kay IP, de Feyter PJ, van Domburg RT, Deshpande NV, Ligthart JM, Gijzel AL, Wardeh AJ, Boersma E, Serruys PW (1999). Remodeling of atherosclerotic coronary arteries varies in relation to location and composition of plaque. Am J Cardiol.

[23] Mintz GS, Painter JA, Pichard AD, Kent KM, Satler LF, Popma JJ, Chuang YC, Bucher TA, Sokolowicz LE, Leon MB (1995). Atherosclerosis in angiographically “normal” coronary artery reference segments: an intravascular ultrasound study with clinical correlations. J Am Coll Cardiol.

[24] Schoenhagen P, Ziada KM, Kapadia SR, Crowe TD, Nissen SE, Tuzcu EM (2000). Extent and direction of arterial remodeling in stable versus unstable coronary syndromes: an intravascular ultrasound study. Circulation.

[25] Okura H, Kataoka T, Matsushita N, Shimeno K, Yoshiyama M, Yoshikawa J, Yoshida K (2013). Culprit lesion remodelling and long-term prognosis in patients with acute coronary syndrome: an intravascular ultrasound study. Eur Heart J Cardiovasc Imaging..

[26] Di Mario C, Görge G, Peters R, Kearney P, Pinto F, Hausmann D, von Birgelen C, Colombo A, Mudra H, Roelandt J, Erbel R (1998). Clinical application and image interpretation in intracoronary ultrasound. Study Group on Intracoronary Imaging of the Working Group of Coronary Circulation and of the Subgroup on Intravascular Ultrasound of the Working Group of Echocardiography of the European Society of Cardiology. Eur Heart J.

[27] Mintz GS, Nissen SE, Anderson WD, Bailey SR, Erbel R, Fitzgerald PJ, Pinto FJ, Rosenfield K, Siegel RJ, Tuzcu EM, American Yock PG (2001). College of cardiology clinical expert consensus document on standards for acquisition, measurement and reporting of intravascular ultrasound studies (IVUS). A report of the American college of cardiology task force on clinical expert consensus documents. J Am Coll Cardiol.

[28] Glagov S, Weisenberg E, Zarins CK, Stankunavicius R, Kolettis GJ (1987). Compensatory enlargement of human atherosclerotic coronary arteries. N Engl J Med.

[29] Jensen LO, Thayssen P, Mintz GS, Maeng M, Junker A, Galloe A, Christiansen EH, Hoffmann SK, Pedersen KE, Hansen HS, Hansen KN (2007). Intravascular ultrasound assessment of remodelling and reference segment plaque burden in type-2 diabetic patients. Eur Heart J.

[30] Nicholls SJ, Tuzcu EM, Kalidindi S, Wolski K, Moon KW, Sipahi I, Schoenhagen P, Nissen SE (2008). Effect of diabetes on progression of coronary atherosclerosis and arterial remodeling: a pooled analysis of 5 intravascular ultrasound trials. J Am Coll Cardiol.

[31] Hong YJ, Jeong MH, Choi YH, Song JA, Ahmed K, Lee KH, Kim DH, Lee MG, Park KH, Sim DS, Yoon NS, Yoon HJ, Kim KH, Park HW, Kim JH, Ahn Y, Cho JG, Park JC, Kang JC (2013). Positive remodeling is associated with vulnerable coronary plaque components regardless of clinical presentation: virtual histology-intravascular ultrasound analysis. Int J Cardiol.

[32] Yamada R, Okura H, Kume T, Saito K, Miyamoto Y, Imai K, Tsuchiya T, Maehama T, Okahashi N, Obase K, Hayashida A, Neishi Y, Kawamoto T, Yoshida K (2010). Relationship between arterial and fibrous cap remodeling: a serial three-vessel intravascular ultrasound and optical coherence tomography study. Circ Cardiovasc Interv..

[33] Kovarnik T, Chen Z, Mintz GS, Wahle A, Bayerova K, Kral A, Chval M, Kopriva K, Lopez J, Sonka M, Linhart A (2017). Plaque volume and plaque risk profile in diabetic vs. non-diabetic patients undergoing lipid-lowering therapy: a study based on 3D intravascular ultrasound and virtual histology. Cardiovasc Diabetol..

[34] Yang DJ, Lee MS, Kim WH, Park HW, Kim KH, Kwon TG, Kim SW, Rihal CS, Lerman A, Bae JH (2013). The impact of glucose control on coronary plaque composition in patients with diabetes mellitus. J Invasive Cardiol..

[35] Okada K, Hibi K, Gohbara M, Kataoka S, Takano K, Akiyama E, Matsuzawa Y, Saka K, Maejima N, Endo M, Iwahashi N, Tsukahara K, Kosuge M, Ebina T, Fitzgerald PJ, Honda Y, Umemura S, Kimura K (2015). Association between blood glucose variability and coronary plaque instability in patients with acute coronary syndromes. Cardiovasc Diabetol..

[36] Kuroda M, Shinke T, Sakaguchi K, Otake H, Takaya T, Hirota Y, Sugiyama D, Nakagawa M, Hariki H, Inoue T, Osue T, Taniguchi Y, Iwasaki M, Nishio R, Kinutani H, Konishi A, Hiranuma N, Takahashi H, Terashita D, Hirata K (2015). Effect of daily glucose fluctuation on coronary plaque vulnerability in patients pre-treated with lipid-lowering therapy: a prospective observational study. JACC Cardiovasc Interv..

[37] Tavares CA, Rassi CH, Fahel MG, Wajchenberg BL, Rochitte CE, Lerario AC (2016). Relationship between glycemic control and coronary artery disease severity, prevalence and plaque characteristics by computed tomography coronary angiography in asymptomatic type 2 diabetic patients. Int J Cardiovasc Imaging.

[38] Yoshida N, Yamamoto H, Shinke T, Otake H, Kuroda M, Terashita D, Takahashi H, Sakaguchi K, Hirota Y, Emoto T, Amin HZ, Mizoguchi T, Hayashi T, Sasaki N, Yamashita T, Ogawa W, Hirata KI (2017). Impact of CD14++ CD16+ monocytes on plaque vulnerability in diabetic and non-diabetic patients with asymptomatic coronary artery disease: a cross-sectional study. Cardiovasc Diabetol..

[39] Watanabe M, Kawai Y, Kitayama M, Akao H, Motoyama A, Wakasa M, Saito R, Aoki H, Fujibayashi K, Tsuchiya T, Nakanishi H, Saito K, Takeuchi M, Kajinami K (2017). Diurnal glycemic fluctuation is associated with severity of coronary artery disease in prediabetic patients: possible role of nitrotyrosine and glyceraldehyde-derived advanced glycation end products. J Cardiol.

[40] Kakuta K, Dohi K, Miyoshi M, Yamanaka T, Kawamura M, Masuda J, Kurita T, Ogura T, Yamada N, Sumida Y, Ito M (2017). Impact of renal function on the underlying pathophysiology of coronary plaque composition in patients with type 2 diabetes mellitus. Cardiovasc Diabetol..

[41] Kim SH, Moon JY, Lim YM, Kim KH, Yang WI, Sung JH, Yoo SM, Kim IJ, Lim SW, Cha DH, Cho SY (2015). Association of insulin resistance and coronary artery remodeling: an intravascular ultrasound study. Cardiovasc Diabetol..

[42] Choi KM, Yoo HJ, Kim HY, Lee KW, Seo JA, Kim SG, Kim NH, Choi DS, Baik SH (2009). Association between endogenous secretory RAGE, inflammatory markers and arterial stiffness. Int J Cardiol.

[43] Aronson D (2003). Cross-linking of glycated collagen in the pathogenesis of arterial and myocardial stiffening of aging and diabetes. J Hypertens.

[44] Tayebjee MH, Lip GY, MacFadyen RJ (2005). What role do extracellular matrix changes contribute to the cardiovascular disease burden of diabetes mellitus?. Diabet Med.

[45] Wohlfarth V, Drumm K, Mildenberger S, Freudinger R, Gekle M (2003). Protein uptake disturbs collagen homeostasis in proximal tubule-derived cells. Kidney Int Suppl.

[46] Kuzuya M, Asai T, Kanda S, Maeda K, Cheng XW, Iguchi A (2001). Glycation cross-links inhibit matrix metalloproteinase-2 activation in vascular smooth muscle cells cultured on collagen lattice. Diabetologia.

[47] Kumeda Y, Inaba M, Shoji S, Ishimura E, Inariba H, Yabe S, Okamura M, Nishizawa Y (2008). Significant correlation of glycated albumin, but not glycated haemoglobin, with arterial stiffening in haemodialysis patients with type 2 diabetes. Clin Endocrinol.

[48] Ren X, Ren L, Wei Q, Shao H, Chen L, Liu N (2017). Advanced glycation end-products decreases expression of endothelial nitric oxide synthase through oxidative stress in human coronary artery endothelial cells. Cardiovasc Diabetol..

[49] Yamada S, Inaba M, Shidara K, Okada S, Emoto M, Ishimura E, Nishizawa Y (2008). Association of glycated albumin, but not glycated hemoglobin, with peripheral vascular calcification in hemodialysis patients with type 2 diabetes. Life Sci.

[50] Nasrallah MM, El-Shehaby AR, Osman NA, Salem MM, Nassef A, El Din UA (2012). Endogenous soluble receptor of advanced glycation end-products (esRAGE) is negatively associated with vascular calcification in non-diabetic hemodialysis patients. Int Urol Nephrol.

[51] Xie Z, Dong N, Sun R, Liu X, Gu X, Sun Y, Du H, Dai J, Liu Y, Hou J, Tian J, Yu B (2017). Relation between baseline plaque features and subsequent coronary artery remodeling determined by optical coherence tomography and intravascular ultrasound. Oncotarget..

[52] Inaba S, Mintz GS, Farhat NZ, Fajadet J, Dudek D, Marzocchi A, Templin B, Weisz G, Xu K, de Bruyne B, Serruys PW, Stone GW, Maehara A (2014). Impact of positive and negative lesion site remodeling on clinical outcomes: insights from PROSPECT. JACC Cardiovasc Imaging..

[53] Hong YJ, Jeong MH, Choi YH, Ko JS, Lee MG, Kang WY, Lee SE, Kim SH, Park KH, Sim DS, Yoon NS, Youn HJ, Kim KH, Park HW, Kim JH, Ahn Y, Cho JG, Park JC, Kang JC (2009). Positive remodeling is associated with more plaque vulnerability and higher frequency of plaque prolapse accompanied with post-procedural cardiac enzyme elevation compared with intermediate/negative remodeling in patients with acute myocardial infarction. J Cardiol.

[54] Okura H, Taguchi H, Kubo T, Toda I, Yoshiyama M, Yoshikawa J, Yoshida K (2007). Impact of arterial remodelling and plaque rupture on target and non-target lesion revascularisation after stent implantation in patients with acute coronary syndrome: an intravascular ultrasound study. Heart.

[55] Wendt TM, Tanji N, Guo J, Kislinger TR, Qu W, Lu Y, Bucciarelli LG, Rong LL, Moser B, Markowitz GS, Stein G, Bierhaus A, Liliensiek B, Arnold B, Nawroth PP, Stern DM, D’Agati VD, Schmidt AM (2003). RAGE drives the development of glomerulosclerosis and implicates podocyte activation in the pathogenesis of diabetic nephropathy. Am J Pathol.

[56] Bucciarelli LG, Wendt T, Qu W, Lu Y, Lalla E, Rong LL, Goova MT, Moser B, Kislinger T, Lee DC, Kashyap Y, Stern DM, Schmidt AM (2002). RAGE blockade stabilizes established atherosclerosis in diabetic apolipoprotein E-null mice. Circulation.

[57] Shoji T, Koyama H, Morioka T, Tanaka S, Kizu A, Motoyama K, Mori K, Fukumoto S, Shioi A, Shimogaito N, Takeuchi M, Yamamoto Y, Yonekura H, Yamamoto H, Nishizawa Y (2006). Receptor for advanced glycation end products is involved in impaired angiogenic response in diabetes. Diabetes.

[58] Tan KCB, Chow WS, Tso AWK, Xu A, Tse HF, Hoo RLC, Betteridge DJ, Lam KSL (2007). Thiazolidinedione increases serum soluble receptor for advanced glycation end-products in type 2 diabetes. Diabetologia.

[59] Carson AP, Muntner P, Selvin E, Carnethon MR, Li X, Gross MD, Garvey WT, Lewis CE (2016). Do glycemic marker levels vary by race? Differing results from a cross-sectional analysis of individuals with and without diagnosed diabetes. BMJ Open Diabetes Res Care..

[60] Brinkley TE, Leng X, Nicklas BJ, Kritchevsky SB, Ding J, Kitzman DW, Hundley WG (2017). Racial differences in circulating levels of the soluble receptor for advanced glycation endproducts in middle-aged and older adults. Metabolism..

